# Cutaneous Manifestation of Eruptive Xanthoma as a Consequence of Severe Hypertriglyceridemia: A Case Report Highlighting Diagnostic and Therapeutic Challenges

**DOI:** 10.7759/cureus.78329

**Published:** 2025-02-01

**Authors:** Jeremi Morka, Aleksandra Morajko, Sylwia Otulak, Karolina Zarańska, Grażyna Wąsik

**Affiliations:** 1 Medicine, University of Opole, Opole, POL; 2 Dermatology, Provincial Hospital of Opole, Opole, POL; 3 Dermatology, University of Opole, Opole, POL

**Keywords:** eruptive xanthomas, hypertriglyceridemia, lipid disorders, lipid-laden foam cells, metabolic syndrome, obesity

## Abstract

Eruptive xanthomas are clinical manifestations of lipid-laden foam cells in the dermis. The morphological subtype and anatomical location of xanthomas can provide insight into the underlying lipid disorder. Hyperlipidemia, a common condition in the general population, is categorized as primary or secondary depending on disruptions in endogenous or exogenous lipoprotein pathways.

We describe the case of a 29-year-old female with severe obesity who presented with numerous yellow papules on the trunk, buttocks, and extensor surfaces of the lower limbs, persisting for three months without subjective symptoms. Examination revealed inflammatory halos around some of the lesions. Dermoscopy showed irregularly shaped, light-yellow areas on a dense, yellowish background. Laboratory investigations revealed extreme hypertriglyceridemia (>4000 mg/dL), elevated total cholesterol (>1000 mg/dL), reduced high-density lipoprotein (HDL) and low-density lipoprotein (LDL) levels and undetectable aminotransferase levels. The patient met the criteria for metabolic syndrome and was newly diagnosed with diabetes mellitus.

A diagnosis of eruptive xanthomas was made, linked to impaired triglyceride clearance and hepatic overproduction of triglyceride-rich lipoproteins secondary to diabetes, obesity, excessive caloric intake, and oral contraceptive use. The Fredrickson classification system identified hypertriglyceridemia as part of types I, IV, and V hyperlipoproteinemia.

This case highlights the significance of a comprehensive diagnostic approach in patients with xanthomas, taking into account associated risk factors, family history, and lipoprotein abnormalities for early detection. Prompt treatment can lead to the complete resolution of eruptive xanthomas and prevent fatal complications such as acute pancreatitis.

## Introduction

Eruptive xanthomas are cutaneous manifestations characterized by lipid-laden foam cells accumulating within the dermis [1]. These lesions often serve as a clinical clue to underlying lipid metabolism disorders. The morphological subtype and anatomical distribution of xanthomas can provide valuable insight into the specific nature of the lipid abnormality, guiding the diagnostic process [2].

Hyperlipidemia, a prevalent condition in the general population, encompasses a wide spectrum of lipid disturbances. It is broadly classified into primary hyperlipidemia, arising from genetic defects in endogenous lipoprotein pathways, and secondary hyperlipidemia, caused by exogenous factors such as diabetes, obesity, or medications. Identifying the precise etiology is essential for targeted management [3]. 

This case report highlights the diagnostic and therapeutic challenges in a patient with severe hypertriglyceridemia manifesting as eruptive xanthomas, underscoring the need for a multidisciplinary approach to optimize outcomes.

## Case presentation

**Figure 1:**
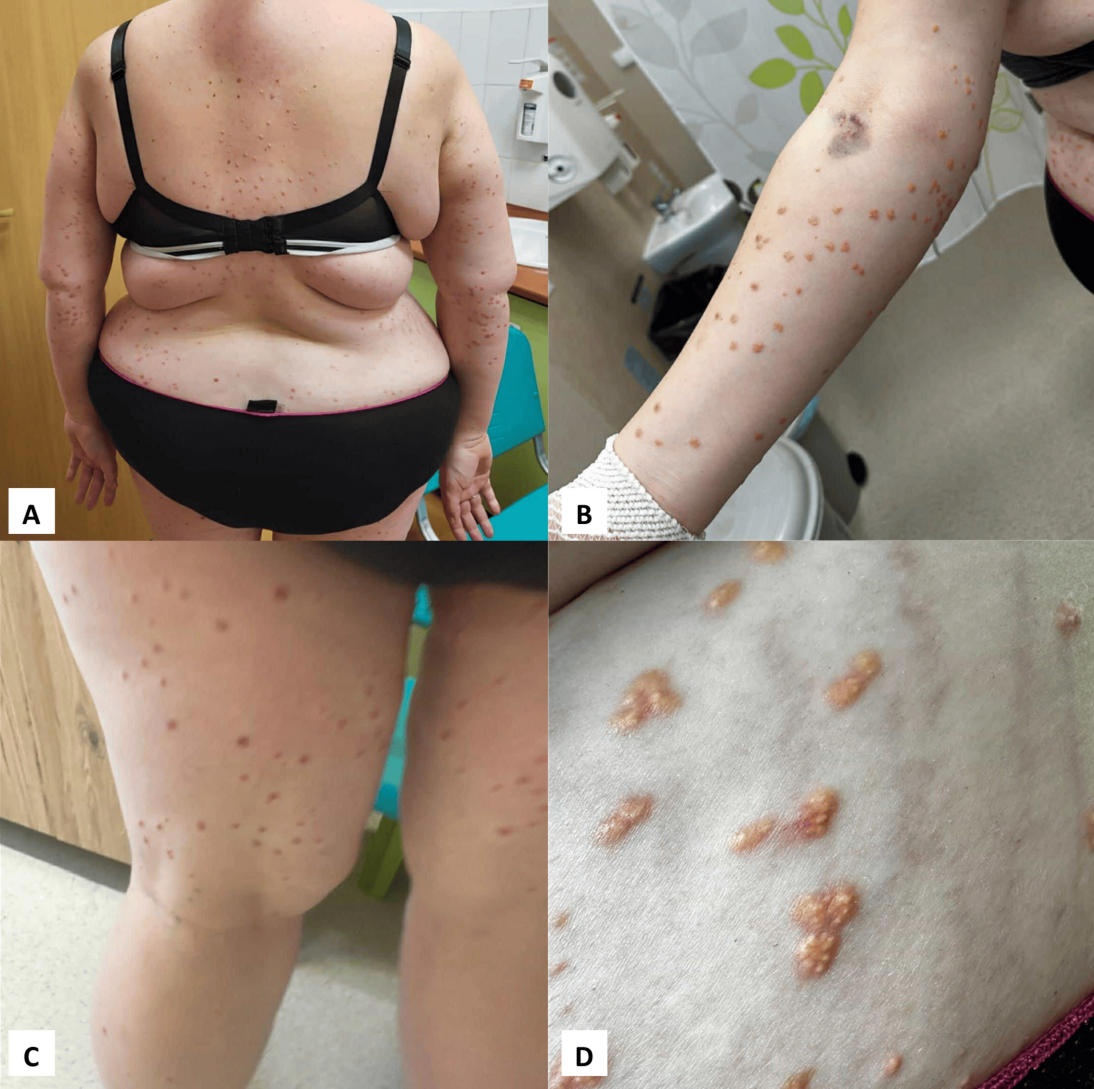
Diffused eruption of papules: lesions on patient's back (A), right forearm (B), tights (C), buttocks (D)

**Figure 2:**
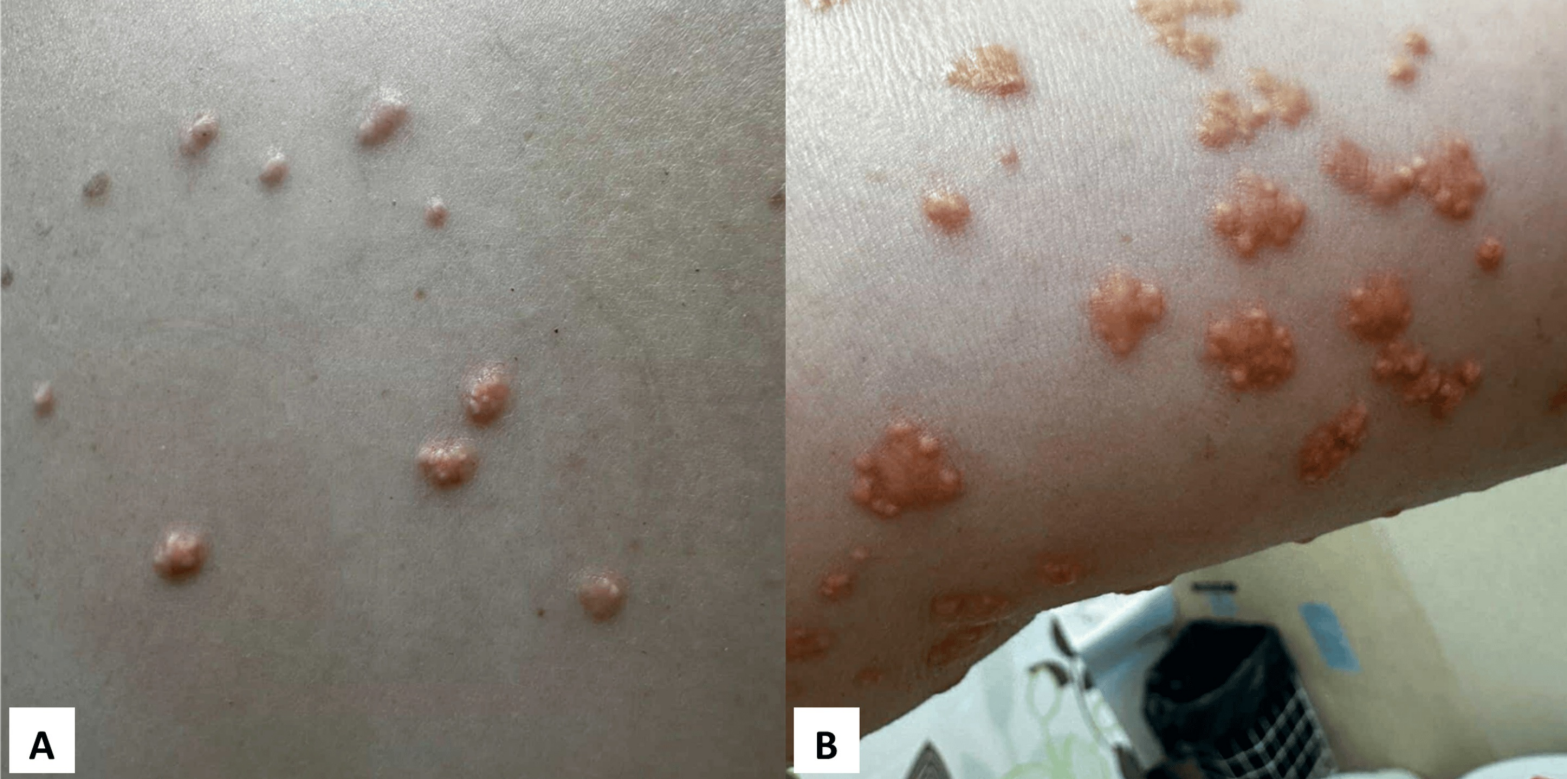
Papules on the skin presented as isolated lesions (A) or in clusters (B)

The following case presents a 29-year-old female patient admitted to the dermatology department due to a diffuse eruption of yellowish papules. The papules had appeared three months earlier, affecting the skin on the trunk, buttocks, upper limbs, and thighs (Figure 1). The papules appeared on the skin either as solitary lesions or clustered (Figure 2), with a white discoloration observed at their apex.

**Figure 3:**
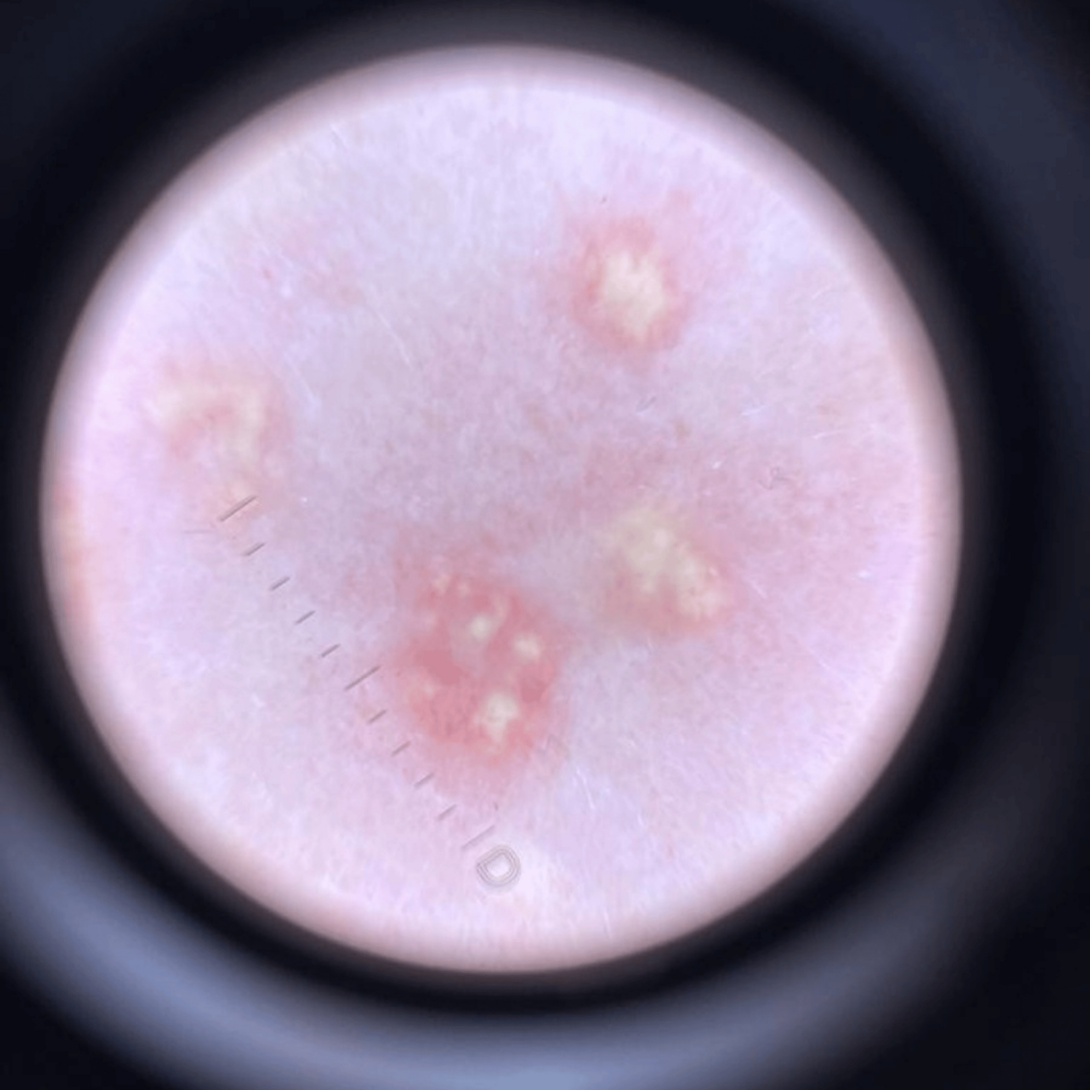
Dermoscopy image of the papules under polarized light

In the physical examination, an inflammatory halo around some of the lesions was noted. The epiluminescence microscopy image revealed asymmetric pale yellow papules, heterogeneous in structure and elevated above the plane of the skin (Figure 3).

The patient reported no somatic symptoms and no history of other concomitant diseases or allergies. Her ongoing medication included oral hormonal contraception. Previous outpatient therapy included oral corticosteroids (prednisone 20 mg/day), second-generation antihistamines (fexofenadine 180 mg/day), and oral antibiotic therapy (amoxicillin + clavulanic acid), all without satisfactory effect.

Laboratory tests showed significantly elevated triglycerides (>4000 mg/dL), total cholesterol (>1000 mg/dL), reduced high-density lipoprotein (HDL) and low-density lipoprotein (LDL) levels, and elevated aminotransferase levels. No renal dysfunction was observed. Additional laboratory findings, including HbA1c (13.4%) and glucose (273 mg/dL), led to a diagnosis of diabetes. No significant deviations in vital signs were observed; blood pressure was 120/70, heart rate 80, and saturation 98%. The waist measured circumference was 161 cm (63.4 inches). The patient was burdened with morbid obesity (BMI 50.5) (Table 1) and met the criteria for the diagnosis of metabolic syndrome (Table 2).

**Table 1:** Laboratory results with reference range HDL - high-density lipoprotein; LDL - low-density lipoprotein

Parameter	Result	Reference range/normal values
Triglycerides	>4000 mg/dl	<150 mg/dl
Total cholesterol	>1000 mg/dl	<200 mg/dl
HDL cholesterol	13.6 mg/dl	>40 mg/dl (men), >50 mg/dl (women)
LDL cholesterol	41.8 mg/dl	<100 mg/dl
Non-HDL Cholesterol	986.4 md/dl	Based on cardiovascular risk: extreme (<70 mg/dL), very high (<85 mg/dl), high (<100 mg/dl), moderate/low (<130 mg/dl)
Alanine transaminase (ALT)	96 U/L	<35 U/L
Aspartate transaminase (AST)	85 U/L	<35 U/L
eGFR	159.1 ml/(min × 1,73 m²)	>60 ml/(min × 1,73 m²)
HbA1c	13.4%	4-5.6% (normal), 5.7-6.4% (pre-diabetes), ≥6.5% (diabetes)
Glucose	273 mg/dl	Fasting: 70-100 mg/dl (normal), >126 mg/dl (diabetes)
Body mass index (BMI)	50.5 kg/m^2^	Normal: 18.5-24.9 kg/m^2^, Overweight: 25-29.9 kg/m^2^, Obese: ≥30 kg/m^2^

**Table 2:** Metabolic syndrome diagnostic criteria Metabolic syndrome is diagnosed in patients with obesity (meeting basic criteria) who fulfill at least two out of three additional diagnostic criteria HDL - high-density lipoprotein; BP - blood pressure

Basic diagnostic criteria (one of the following required)	
Abdominal obesity	Waist circumference: ≥ 88 cm (women) or ≥ 102 cm (men)
Body mass index	≥ 30 kg/m²
Additional diagnostic criteria (at least two of three required)	
Prediabetes or diabetes	Fasting glucose ≥100 mg/dL
	Glucose ≥140 mg/dL after 120 min in oral glucose tolerance test
	HbA1c ≥ 5.7%
	patient on glucose-lowering medication
Elevated non-HDL cholesterol	Non-HDL cholesterol ≥ 130 mg/dL
	On lipid-lowering treatment
High normal blood pressure or hypertension	Systolic BP ≥ 130 and/or diastolic BP ≥ 85 mmHg (in-office measurement)
	Systolic BP ≥ 130 and/or diastolic BP ≥ 80 mmHg (ambulatory measurement)
	Patient on anti-hypertensive treatment

The diagnosis of eruptive xanthomas was subsequently made as a cutaneous manifestation of hypercholesterolemia with hypertriglyceridemia. Treatment with statins (rosuvastatin 40 mg/day) and fibrates (fenofibrate 267 mg/day) was initiated. Hypoglycemic agents therapy, including sodium-glucose transport protein 2 (SGLT-2) inhibitor (empagliflozin 10mg/day), metformine 500mg/day, sulfonylurea (gliclazide 60 mg/day), a long-acting modified form of insulin (insulin glargine 8-10 units/day) was also incorporated into the treatment plan. The patient was advised to reduce her intake of high-saturated fatty acids and trans-unsaturated fatty acids, as well as increase her dietary fiber intake. In order to decrease lipid disturbance, the reduction of excess body weight, alcohol intake, and the consumption of monosaccharides and disaccharides were recommended as a part of non-pharmacological management. All the measures taken resulted in the partial disappearance of skin lesions and aimed to prevent life-threatening complications of lipid imbalance, such as acute pancreatitis. The patient was urgently referred to the diabetes department for treatment of unregulated diabetes. Furthermore, she was advised to remain under close supervision at the dietetic clinic. The patient failed to attend the designated ward, and no subsequent follow-up appointments were recorded.

## Discussion

Lipid disorders are among the most common risk factors for cardiovascular disease, both in Poland and worldwide. Dyslipidemia, characterized by irregular lipid levels in the bloodstream, plays a crucial role in the progression of atherosclerosis, which can lead to diseases such as coronary artery disease, heart attacks, and strokes [4]. A Polish study, LIPIDOGRAM2015, by Studziński et al. found that dyslipidemia was present in over 84% of the studied population, highlighting its role as a predominant cardiovascular risk factor in Poland [5]. Xanthomas, particularly eruptive xanthomas, can be an early sign of lipid disorders, especially severe hypertriglyceridemia. Eruptive xanthomas are small, yellowish, dome-shaped papules that occur in about 8.5% of patients with severe hypertriglyceridemia [6]. This condition is often linked to triglyceride levels exceeding 2000 mg/dL, with more severe cases reaching higher numbers, far above the normal range of less than 150 mg/dL [7]. The overall incidence of eruptive xanthomas is estimated to be approximately 18 cases per 100,000 individuals [8,9]. These papules, typically measuring 1-4 mm in diameter, are most commonly found on the extensor surfaces of the limbs, hands, and buttocks [10].

Histological analysis reveals that eruptive xanthomas consist of keratinized, multilayered squamous epithelium overlying dermal foam cells, which are macrophages engorged with lipids [11]. These lesions are commonly linked to lipid metabolism disorders, but they are not solely indicative of hypertriglyceridemia. The specific location and pattern of the lesions can offer valuable diagnostic insights into the underlying lipid condition. The diagnosis of eruptive xanthoma is primarily based on its characteristic clinical presentation, dermoscopic findings, and laboratory abnormalities. In cases of diagnostic uncertainty, histopathological examination remains the gold standard for confirming the presence of foam cells in the dermis [12].

In the differential diagnosis of eruptive xanthomas, conditions such as molluscum contagiosum, histiocytosis, and generalized granuloma annular should be considered. These alternative diagnoses are particularly relevant in cases where triglyceride levels are not significantly elevated [13,14]. Xanthomas are also associated with hematological malignancies. The presence of Eruptive xanthoma in the absence of hypertriglyceridemia may be considered as a paraneoplastic syndrome. The diagnosis of xanthoma consequently, may be regarded as a significant factor in the further diagnosis of neoplastic diseases of the hematopoietic system [15].

Initial management focuses on lifestyle modification, including dietary restrictions to limit carbohydrate and saturated fat intake, as well as achieving weight reduction. Elevated triglyceride levels are most often lowered using fibric acid derivatives and omega-3 fatty acids. In situations where rapid recovery is essential, therapeutic plasma exchange (TPE), a process that removes excess lipids from the blood, can be employed [16].

Starting hypolipidemic treatment early is essential for speeding up the resolution of eruptive xanthomas and greatly lowering the chances of severe complications like acute pancreatitis and cardiovascular events, both linked to hypertriglyceridemia. Timely identification and treatment of eruptive xanthomas can help reduce the morbidity and mortality associated with acute pancreatitis [17].

When pharmacological treatments fail to achieve desired outcomes, procedural options like laser therapy or cryosurgery can be explored for addressing persistent lesions. For example, ablative lasers such as CO₂ and Er:YAG have been employed to treat xanthelasma palpebrarum - a related condition - by effectively targeting perivascular foam cells and coagulating dermal vessels through rapid heating and vaporization of intracellular water [18]. Similarly, cryotherapy using liquid nitrogen has shown potential as a convenient and low-risk method for treating xanthelasma [19]. These procedures offer alternative solutions when medication alone does not completely resolve the condition. 

Identifying xanthomas as an early indicator of lipid disorders is essential for timely diagnosis and intervention. Managing the underlying hypertriglyceridemia not only alleviates the skin lesions but also reduces the risk of serious, life-threatening conditions. This underscores the importance of a comprehensive, multidisciplinary treatment approach involving dermatologists, cardiologists, and primary care providers [20].

## Conclusions

The presented case highlights the significance of a well-executed diagnostic process in patients presenting symptoms of eruptive xanthoma. To make a prompt and accurate diagnosis, it is important to distinguish between potential factors contributing to the lipid disorder. These include the age of onset of symptoms, a comprehensive family history, and any specific abnormalities in lipoprotein metabolism. Evaluating these elements is crucial to identifying the underlying cause and determining the most effective treatment strategy.
